# Editorial: Early detection and intervention for unilateral hearing loss and mild bilateral hearing loss in children: clinical practices and outcomes

**DOI:** 10.3389/fped.2024.1400074

**Published:** 2024-04-10

**Authors:** Teresa Y. C. Ching, Elizabeth M. Fitzpatrick, Kerttu Huttunen, Ulrika Löfkvist, Carmen Kung, Valerie Sung

**Affiliations:** ^1^ NextSense Institute, NextSense, Sydney, NSW, Australia; ^2^ Macquarie School of Education, Macquarie University, Sydney, NSW, Australia; ^3^ School of Health and Rehabilitation Science, University of Queensland, Brisbane, QLD, Australia; ^4^ Faculty of Health Sciences, University of Ottawa, Ottawa, ON, Canada; ^5^ Child Hearing Laboratory, CHEO Research Institute, Ottawa, ON, Canada; ^6^ Research Unit of Logopedics, Faculty of Humanities, University of Oulu, Oulu, Finland; ^7^ Department of Otorhinolaryngology, Head and Neck Surgery, Oulu University Hospital, Oulu, Finland; ^8^ Medical Research Center Oulu, University of Oulu, Oulu, Finland; ^9^ Department of Public Health and Caring Sciences, Uppsala University, Uppsala, Sweden; ^10^ Department of Clinical Service, Intervention and Technology (CLINTEC), Karolinska Institute, Stockholm, Sweden; ^11^ Department of Linguistics, Macquarie University, Sydney, NSW, Australia; ^12^ Prevention Innovation, Population Health, Murdoch Children’s Research Institute, Parkville, VIC, Australia; ^13^ Centre for Community Child Health, Royal Children’s Hospital, Parkville, VIC, Australia; ^14^ Department of Pediatrics, University of Melbourne, Parkville, VIC, Australia

**Keywords:** unilateral hearing loss (UHL), mild bilateral hearing loss, children, clinical practices, outcomes

**Editorial on the Research Topic** Early detection and intervention for unilateral hearing loss and mild bilateral hearing loss in children: clinical practices and outcomes

Most newborn hearing screening (NHS) programs aim to identify children with permanent moderate to profound bilateral hearing loss. In addition to these children, NHS programs may identify children with unilateral hearing loss (UHL) and those with mild bilateral hearing loss (MBHL). Previously, these children have typically been identified at 4–5 years of age. The very early identification of children with UHL raises questions about early management, particularly about early provision of amplification for which there is no clear evidence. A similar lack of evidence for the efficacy of intervention exists for children with MBHL. The contributions to this special issue reflect state-of-the-art evidence on clinical practices and outcomes of children who are impacted by a hearing loss during an important developmental period, especially for speech, language, listening effort, emotions and behaviour, and quality of life.

Early detection has provided an opportunity to better map the audiologic characteristics and trajectory of hearing loss in children with UHL. This is the focus of two papers in this issue, which reported findings from studies in Australia and Canada. Zhang et al. provided detailed audiologic and clinical profiles of 91 children with congenital UHL. The authors drew attention to the challenge of obtaining reliable behavioral audiologic profiles because despite early diagnosis at an average of 2.1 months, almost half of the children were over age 3 years before the first reliable audiogram was obtained. This study also showed that 78% of children showed deterioration in hearing by the time of their first behavioral audiogram (average age 3 years). Notably, 73% of the children with deterioration progressed to severe to profound hearing loss. In the second paper, Fitzpatrick et al. also undertook an in-depth examination of the trajectory of unilateral hearing loss. Almost half of 177 children followed for an average of 58.9 months experienced further deterioration in hearing including 12% who developed bilateral hearing loss. These reports underscore the importance of careful monitoring of even very mild degrees of UHL, given that 2–3 of every 4 children appear to be at risk for further hearing deterioration in one or both ears.

Other challenges in clinical management of UHL are reported in two papers. Horrocks et al. study showed that children admitted to neonatal intensive care units with UHL, when compared to matched controls without hearing loss, were more likely to have congenital anomalies, developmental impairments and requirements for speech and language therapy. The authors highlighted the need for screening for this group of children because many of the congenital anomalies were not detected at birth, including genetic and clinical follow-up. Patel et al. showed that the lack of evidence in guiding early management of UHL has resulted in much of the decisions on trialing hearing devices being parent- or child-led rather than clinician-led. Of the children who were fitted with hearing devices in the reported cohort, most occurred late (mean age 4.7 years). The authors also highlighted the lack of funding for support services and cochlear implants, despite 28.5% of the families reporting concerns around their care.

The presence of hearing loss reduces auditory input, potentially affecting outcomes. Three studies reported on psychosocial, language and quality-of-life outcomes. Ong et al. showed that school-aged children with UHL and MBHL were just as likely as those with moderate to profound hearing loss to experience more emotional/behavioural difficulties, poorer health-related quality of life, and higher distress reported in their parents compared to population norms. Carew et al. showed that, on average, children with MBHL had poorer language outcomes than those with UHL, and both groups had lower scores compared to population norms. The total health-related quality of life scores were, on average, similar between UHL and MBHL groups. On the other hand, Cupples et al. found that children with congenital UHL had language, functional performance, speech intelligibility and quality-of-life outcomes similar to population norms, but passage comprehension and speech perception in noise were significantly below the typical range. They identified a relationship between better nonverbal cognitive ability and language results, which underscored the importance of examining cognitive ability in future studies.

Binaural processing can help listeners locate sound sources and improve their ability to hear and understand target speech in noisy environments. As these benefits relied on combining auditory inputs from both ears, they are lessened when hearing is reduced in one or both ears. Two studies explored the consequences of decreased audibility. Lewis et al. reported that children with untreated UHL or MBHL located talkers more easily and achieved better speech perception in noise when assessed in the audio-visual condition than in the auditory-only condition. On average, children with UHL exhibited better speech perception than children with MBHL, but both groups performed more poorly than peers with typical hearing. Dahlgren et al. showed that children with unilateral aural atresia experienced difficulties in localisation, with performance inversely related to degree of hearing thresholds in the atretic ear. However, early aiding with bone conduction hearing aids had mixed effects. A further consequence of decreased audibility is listening-related fatigue, which may underpin problems experienced by listeners with hearing loss. Adams et al. described fatigue experienced by 6- to 16-year-old children with and without hearing loss. Compared with children with typical hearing, listening can cause more fatigue not only for children with bilateral hearing loss but also for children with UHL. This calls for improving the acoustics of learning environments for children.

The articles in this research topic exemplify the diverse outcomes of contemporary cohorts of children with UHL and MBHL, part of which may be attributed to the lack of evidence-based guidelines for management. Despite an unsuccessful attempt to conduct a randomized control trial of amplification for children with MBHL by Sung et al. they shared learnings on engaging families in trials that might generate high-quality evidence. Cupples et al. called for research to evaluate the fitting of hearing devices using random assignment to avoid any confounding influence of degree of hearing loss or past/current level of progress. More research is needed to understand factors influencing the somewhat atypical speech, language and psychosocial outcomes. Given the heterogeneity of children with UHL or MBHL, and the clear risk of progression of hearing loss, it is crucial to develop tailored intervention options and evidence-based guidelines for management.

